# Supplementary material for: Angelica sinensis polysaccharide as potential protectants against recurrent spontaneous abortion: focus on autophagy regulation
Source: Front Med (Lausanne). 2025 Jan 15;12:1522503. doi: 10.3389/fmed.2025.1522503 (PMC11774876; doi:10.3389/fmed.2025.1522503)
Supplement: Supplementary material S1 — The main instruments used during the LC-MS process, along with their models/specifications and manufacturers. [file Supplementary_file_1.zip › Metabolomics sequencing data FC1.2/8.代谢通路分析/Exposure_Ctrl/all/KEGG/mmu00120.html]

KEGG PATHWAY: Primary bile acid biosynthesis - Mus musculus (house mouse)


# Primary bile acid biosynthesis - Mus musculus (house mouse)


[
Pathway menu
| Organism menu
| Pathway entry
| Show description
| Download
| Help
]

Bile acids are steroid carboxylic acids derived from cholesterol in vertebrates. The primary bile acids, cholic acid and chenodeoxycholic acid, are synthesized in the liver and conjugated with taurine or glycine before secretion via bile into the intestine. The conversion from cholesterol to cholic and chenodeoxycholic acids involves four steps: 1) the initiation of synthesis by 7alpha-hydroxylation of sterol precursors, 2) further modifications to the ring structures, 3) side-chain oxidation and shortening (cleavage) by three carbons, and 4) conjugation of the bile acid with taurine or glycine.


##### Option

Scale:


100%

Image resolution:


 High

##### Search

##### ID search

##### Color

##### Module

Complete only
Including 1 block missing
Including any incomplete

- Pathway modules
  - Lipid metabolism
    - Sterol biosynthesis
      - M00104
        Bile acid biosynthesis
      - M00106
        Conjugated bile acid biosynthesis
      - M00862
        beta-Oxidation, peroxisome


KGML

Image (png) file 1x
